# Leveraging Image Analysis for High-Throughput Plant Phenotyping

**DOI:** 10.3389/fpls.2019.00508

**Published:** 2019-04-24

**Authors:** Sruti Das Choudhury, Ashok Samal, Tala Awada

**Affiliations:** ^1^School of Natural Resources, University of Nebraska-Lincoln, Lincoln, NE, United States; ^2^Department of Computer Science and Engineering, University of Nebraska-Lincoln, Lincoln, NE, United States; ^3^Agricultural Research Division, University of Nebraska-Lincoln, Lincoln, NE, United States

**Keywords:** high-throughput plant phenotyping, image analysis, multimodal image sequence, phenotype taxonomy, structural phenotype, physiological phenotype, temporal phenotype

## Abstract

The complex interaction between a genotype and its environment controls the biophysical properties of a plant, manifested in observable traits, i.e., plant's phenome, which influences resources acquisition, performance, and yield. High-throughput automated image-based plant phenotyping refers to the sensing and quantifying plant traits non-destructively by analyzing images captured at regular intervals and with precision. While phenomic research has drawn significant attention in the last decade, extracting meaningful and reliable numerical phenotypes from plant images especially by considering its individual components, e.g., leaves, stem, fruit, and flower, remains a critical bottleneck to the translation of advances of phenotyping technology into genetic insights due to various challenges including lighting variations, plant rotations, and self-occlusions. The paper provides (1) a framework for plant phenotyping in a multimodal, multi-view, time-lapsed, high-throughput imaging system; (2) a taxonomy of phenotypes that may be derived by image analysis for better understanding of morphological structure and functional processes in plants; (3) a brief discussion on publicly available datasets to encourage algorithm development and uniform comparison with the state-of-the-art methods; (4) an overview of the state-of-the-art image-based high-throughput plant phenotyping methods; and (5) open problems for the advancement of this research field.

## 1. Introduction

The temporal variation in the plant's morphological and functional traits regulated by genotype and the environment plays a crucial role for the development of crops that impact both yield and quality (Lobos et al., 2017). High-throughput image-based plant phenotyping facilitates the computation of phenotypes by analyzing a large number of plants in short time interval with precision, nullifying the need for time-consuming physical human labor (Das Choudhury et al., 2018). The process is generally non-destructive, allowing the same traits to be quantified repeatedly at multiple times and scales during a plant's life cycle. It is an interdisciplinary research field involving computer science, biology, remote sensing, statistics, and genomics in the effort to link intricate plant phenotypes to genetic expression in order to meet current and emerging issues in agriculture relating to future food security under dwindling natural resources and projected climate variability and change.

Plants are not static but living organisms with constantly increasing complexity in shape, architecture, and appearance. Many plants alter leaf positioning (i.e., phyllotaxy) in response to light signals perceived through the photochrome in order to optimize light interception (Maddonni et al., 2002). In addition to variation in phyllotaxy, growth of individual leaves leads to self-occlusions and leaf crossovers over time posing challenges to image-based plant phenotyping analysis. Other challenges include variation in illumination, cluttered background, and change in zoom levels in the cameras often used to adjust for plant growth.

The state-of-the-art computer vision based approaches mainly focus on the structural and morphological aspects of a plant for computing 2D and 3D phenotypes. However, quantification of physiological processes in plant components, i.e., leaves, stem segments, flowers, fruits, etc., may show differential behavior as a function of environmental stress, and result in different leaf-level photosynthetic activity or carbohydrate content in a plant segment. Similarly, dynamic event-based phenotypes, i.e., identifying important events in plant life cycle, e.g., emergence timing of a new leaf, automated germination detection, timing of emergence detection of fruits and flowers, may provide important insights into the acclimation and adaptation strategies of plants. Thus, we present a novel taxonomy, beyond the well-studied class of structural phenotypes, to illustrate the vast potential of image analysis based methods to assist in understanding of insightful physiological and temporal phenotypes. To achieve this, full range of available imaging modalities and mechanisms must be used. Therefore, we propose a general computational framework for image-based plant phenotyping. We also summarize state-of-the-art image-based plant phenotyping methods with discussion on potential future developments in this field. To systematically evaluate and uniformly compare the methods, benchmark datasets are indispensable. Thus, the paper also provides a comprehensive summary of the publicly available datasets.

## 2. High-Throughput Plant Phenotyping Platform

**Figure 1:**
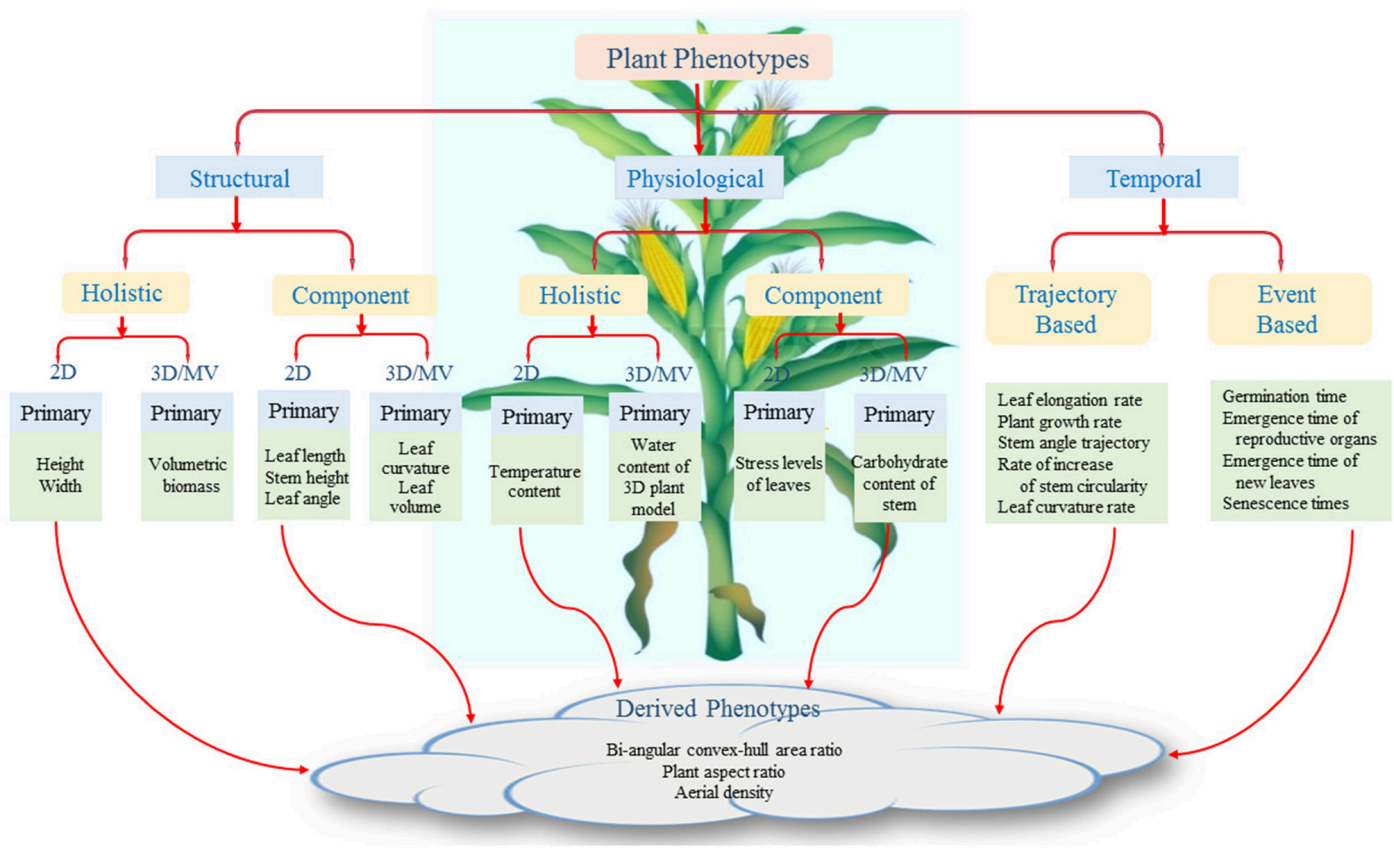
A taxonomy of phenotypes. Key: "MV"-multi-view.

Figure 1 shows a schematic diagram for image-based high-throughput plant phenotyping platform. It analyses temporal image sequences of a group of plants (belonging to different genotypes) captured by multimodal cameras, i.e., visible light, fluorescent, near infrared, infrared and hyperspectral, from different viewing angles. The plants are imaged at regular intervals under various environmental conditions, e.g., drought, salinity, and thermal (Das Choudhury et al., 2016). Motivated by the high-throughput plant phenotyping platform presented in Fahlgren et al. (2015), we design a more advanced representation to explore computer vision based plant phenotyping algorithms in multiple dimensions, i.e., multimodal, multiview and temporal, regulated by genotypes under various environmental conditions. The image sequences captured by visible light camera are often used to compute structural or morphological phenotypes. Fluorescent, near infrared and infrared images are respectively used to analyze the chlorophyll, water, and temperature content of the plants. Thermal infrared imaging is used as proxy for a plant's temperature to detect differences in stomatal conductance as a measure of the plant response to the water status and transpiration rate for abiotic stress adaptation (Lei et al., 2014b). Hyperspectral imaging is uniquely suited to provide insights into the functional properties of plants, e.g., leaf tissue structure, leaf pigments, and water content (Mahlein et al., 2011) and stress resistance mechanisms (Wahabzada et al., 2016). The high-throughput plant phenotyping platforms presented by Rahaman et al. (2015) and Araus and Cairns (2014) provide emphasis on bridging gap between phenotype-genotype relationship from the molecular point of view.

## 3. A Taxonomy for Plant Phenotypes

**Figure 2:**
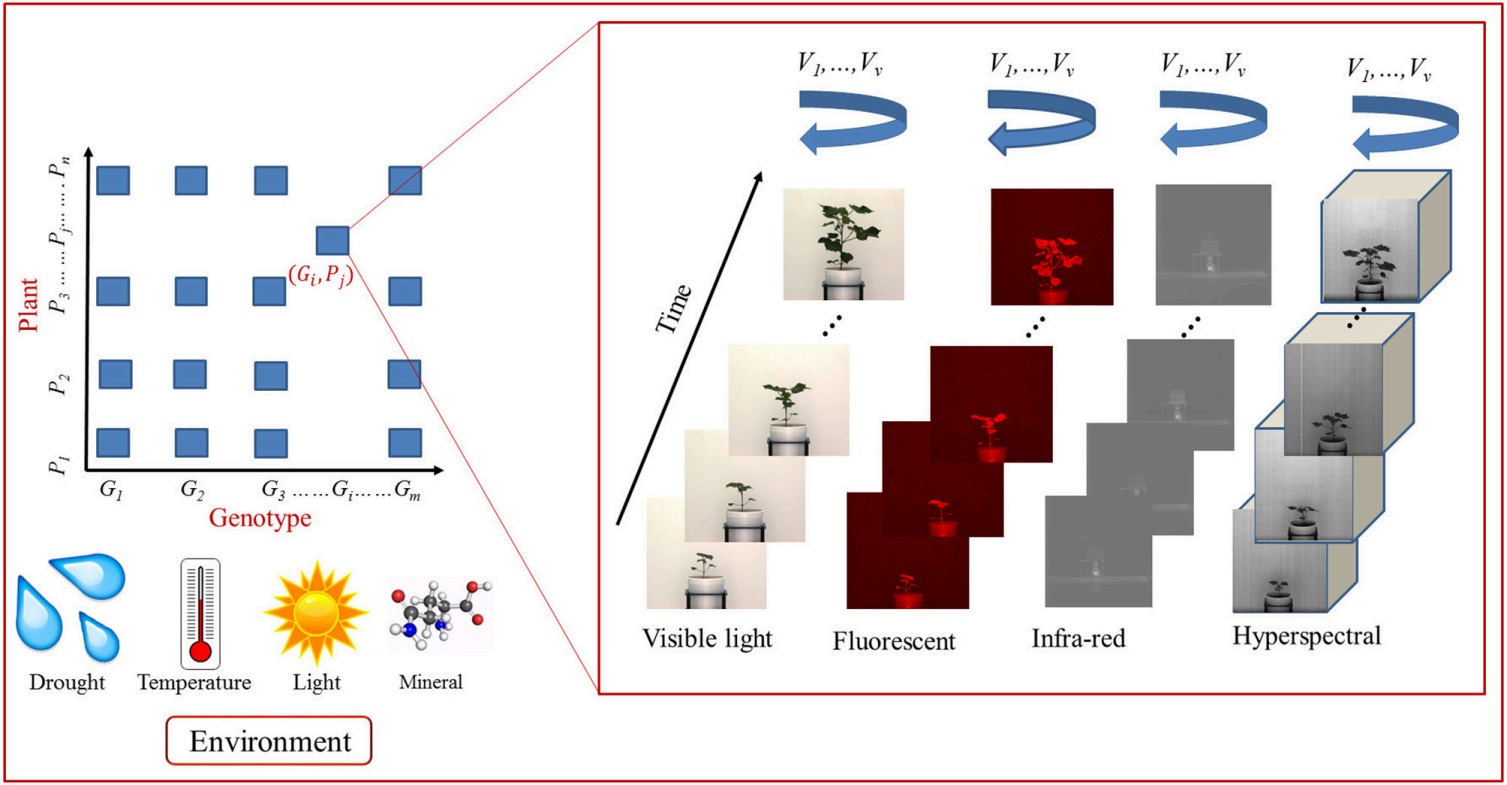
High-throughput plant phenotyping platform.

We present a plant phenotypic taxonomy (Figure 2) which reflects plant phenotypes introduced in recent literature as well as the new challenges that require future research attention. The figure shows that the aboveground plant phenotypes are broadly classified into three categories, namely, structural, physiological, and temporal. The structural phenotypes refer to the morphological attributes of the plants, whereas the physiological phenotypes are related to traits that affect plant processes regulating growth and metabolism. Structural and physiological phenotypes are further divided into two groups: holistic and component. The holistic phenotypes consider the whole plant as a single object and compute its basic geometrical properties, e.g., height of the bounding rectangle to account for plant height, area of the convex-hull to account for plant size (Das Choudhury et al., 2017, 2018). Component phenotypes are computed by considering individual components of the plants, i.e., leaves, stem, flower, and fruit. Examples of component phenotypes include leaf length, chlorophyll content of each leaf, stem angle, flower size, and fruit volume (Gage et al., 2017; He et al., 2017; Das Choudhury et al., 2018; Yin et al., 2018; Zhou et al., 2019).

The different parts of plants grow non-uniformly over space and time. This non-uniformity in growth is also regulated by the genotypes. The temporal phenotypes are computed by analyzing a sequence of images. We propose two different types of temporal phenotypes: trajectory-based and event-based. The structural and physiological phenotypes can be computed from a single image or a sequence of images to take into account of temporal phenotypic characteristics for better genetic variability, e.g., plant growth rate, leaf elongation rate, and trajectories of stem angle (Das Choudhury et al., 2016, 2018; Sun et al., 2018). It includes change in phenotypic traits as a function of time, time-rate of change in stem width and relative growth rates of different leaves. Event-based phenotypes are based on specific events in a plant's life cycle, e.g., germination, emergence of a new leaf, flowering, etc. The timing of the events with reference to an origin (say planting date) is important in understanding a plant's behavior (Agarwal, 2017). While the primary phenotypes refer to one specific characteristic of a plant, derived phenotypes are composed of two or more primary phenotypes, i.e., bi-angular convex-hull area ratio to account for phyllotaxy and plant aspect ratio to provide information on canopy architecture (Das Choudhury et al., 2016).

## 4. Public Datasets

Development and public dissemination of datasets are vital for research in image based plant phenotyping as it provides the broader computer vision research community access to datasets that they typically do not have the ability to generate. Also, standard datasets provide a common basis to compare the performance of plant phenotyping algorithms. We briefly summarize the datasets below that are publicly disseminated.

- **Leaf segmentation challenge (LSC) dataset:** LSC dataset is released to advance the state-of-the-art in leaf segmentation, counting, and tracking of rosette plants. The dataset consists of images of two plant species, i.e., Arabidopsis (*Arabidopsis thaliana*) and tobacco (*Nicotiana tabacum*) organized into three subsets. Subsets A1 (Ara2012) and A2 (Ara2013) consist of top-view time-lapse images of *Arabidopsis thaliana* rosettes. The total number of images in Ara2012 and Ara2013 are 150 and 5048, respectively. Subset A3 (Tobacco) consists of top-view stereo image sequences of tobacco *(Nicotiana tabacum)* plants captured hourly for 30 days. The LSC dataset is publicly available from http://www.plant-phenotyping.org/CVPPP2014-challenge.
- **Michigan State University Plant imagery dataset (MSU-PID):** The MSU-PID dataset (Cruz et al., 2016) consists of images of Arabidopsis (total 2160 × 4 images) and bean (total 325 × 4 images) captured with four types of calibrated cameras, i.e., fluorescent, infrared, RGB color and depth sensor to facilitate research in leaf segmentation, leaf counting, leaf alignment, leaf tracking, and 3D leaf reconstruction. A subset (576 × 4 Arabidopsis images and 175 × 2 bean images) is annotated to provide ground-truth for leaf tip location, leaf segmentation, and leaf alignment. MSU-PID dataset is publicly available from http://cvlab.cse.msu.edu/multi-modality-imagery-database-msu-pid.html Cruz et al. (2016).
- **Panicoid Phenomap-1:** To stimulate the development and evaluation of holistic phenotypes of panicoid grain crops, a public dataset called Panicoid Phenomap-1 is introduced in Das Choudhury et al. (2016). It consists of visible light image sequences of 40 genotypes including at least one representative accession from five panicoid grain crops: maize, sorghum, pearl millet, proso millet, and foxtail millet. The images are captured by the Lemnatec scanalyzer high-throughput plant phenotyping facility at the University of Nebraska-Lincoln (UNL), USA.
- **University of Nebraska-Lincoln Component Plant Phenotyping Dataset (UNL-CPPD):** A benchmark dataset called UNL-CPPD in introduced in Das Choudhury et al. (2018), to spur research in leaf detection and tracking, leaf segmentation, evaluation of holistic, and component phenotypes for maize and cereal crops with similar architecture, e.g., sorghum. UNL-CPPD includes human-annotated ground-truth along with the original image sequences to facilitate image-based component phenotyping analysis.
- **Komatsuna Dataset:** Komatsuna Dataset (Uchiyama et al., 2017) contains images of early growth stages of Komatsuna plants with a leaf annotation tool to facilitate 3D plant phenotyping analysis such as leaf segmentation, tracking, and reconstruction. A set of 5 Komatsuna plants are imaged every 4 h for 10 days using a RGB camera (Multiview dataset) and a RGB camera fitted with structured light depth camera (RGB-D dataset). The dataset is freely available from http://limu.ait.kyushu-u.ac.jp/~agri/komatsuna/.
- **University of Nebraska-Lincoln 3D Plant Phenotyping Dataset (UNL-3DPPD):** This dataset consists of images of 20 maize and 20 sorghum plants for 10 side views to facilitate 3D plant phenotyping research. Plants were imaged once per day using the visible light camera of the UNL Lemnatec Scanalyzer 3D high-throughput phenotyping facility. Panicoid Phenomap-1, UNL-CPPD, and UNL-3DPPD can be freely downloaded from http://plantvision.unl.edu/.
- **Deep Phenotyping Dataset:** This dataset consists of 22 successive top-view image sequences of four accessions of Arabidopsis, i.e., Sf-2, Cvi, Landsberg, and Columbia, captured once daily to study temporal phenotypes for accession classification using convolutional neural network (CNN), recurrent neural network and long-short term memory (LSTM) (Taghavi Namin et al., 2018). The dataset is augmented by rotating each image by 90°, 180°, and 270° to avoid overfitting while training CNN. It can be freely downloaded from https://figshare.com/s/e18a978267675059578f.

## 5. Recent Advancements in Image-Based Plant Phenotyping

This section provides a brief description of the state-of-the-art methods to compute phenotypes from images organized using our taxonomy, i.e., structural, physiological, and temporal.

### 5.1. Structural Phenotypes

#### 5.1.1. 2D Phenotypes

The method in Dellen et al. (2015) uses a leaf-shape model to detect each leaf of a tobacco plant. A graph-based tracking algorithm is used to track the detected leaves in a sequence to measure growth rate as a trajectory-based structural phenotype. Leaf alignment and tracking are formulated as two optimization problems in Yin et al. (2018) based on Chamfer matching and leaf template transformation from fluorescent videos for application in leaf-level photosynthetic capability estimation. Das Choudhury et al. (2018) introduced a set of new holistic and component phenotypes computed from 2D side view image sequences of maize plants, and demonstrated the temporal variations of these phenotypes regulated by genotypes. The method accepts plant image sequence as the input and produces a leaf status report containing the phenotypic information, i.e., the emergence timing, total number of leaves present at any point of time, total number of leaves emerged, the day on which a particular leaf stopped growing or lost, and the length and relative growth rate of individual leaves. The method in Das Choudhury et al. (2017) introduces an algorithm to compute stem angle, a potential measure for plants' susceptibility to lodging, based on graph-based plant architecture determination. A time series clustering analysis is used to summarize the temporal patterns of the stem angles into different groups to provide further insight into genotype specific behavior of the plants. Unlike the methods by Das Choudhury et al. (2016, 2018) which focus on vegetative stage phenotyping analysis of maize, the method by Brichet et al. (2017) develops a robot-assisted imaging pipeline to track the growths of ear and silks based on an ear detection algorithm. The genotypic variation in silk growth rate under drought stress is experimentally demonstrated.

It has been suggested that future progress in image-based plant phenotyping will require a combined effort in the domains of image processing for feature extraction and machine learning for data analysis (Tsaftaris et al., 2016). In recent times, machine learning techniques have gained popularity in high-throughput 2D phenotyping, e.g., in detection of branch shaking locations for robotic cherry harvesting (Amatya et al., 2017) and plant growth analysis (Navarro et al., 2016). Deep learning is an emerging field that promises unparalleled results on many data analysis problems. Building on artificial neural networks, deep approaches have many more hidden layers in the network, and hence have greater discriminative and predictive power. A deconvolutional network is used for segmenting the rosette leaves from the background, and then a CNN is used for leaf counting in the method by Aich and Stavness (2017). The method by Atanbori et al. (2018) re-architects four existing deep neural networks to create “Lite" CNN models in an attempt to reduce their parameters while avoiding overfitting for cost-effective solutions in plant phenotyping. The method also introduces a global hyper-parameter for efficient trade-off between parameter size and accuracy of “Lite" CNN models. The method by Pound et al. (2017) uses CNN to identify quantitative trait loci by classifying biologically relevant features such as root tips, leaf and ear tips, and leaf bases to determine root and shoot architecture.

An open source software tool based on CNN called Deep Plant Phenomics is proposed in Ubbens and Stavness (2017) to compute complex phenotypes from plant image sequences. ResNet50, a deep residual neural network, is used in Dobrescu et al. (2017) as a leaf prediction model to count the number of leaves of the rosette plants. In this method, leaf counting is modeled as a direct regression problem. A comprehensive summary of deep learning algorithms for identification, classification, quantification, and prediction of plant stress phenotypes is presented by Singh et al. (2018). Deep learning has applications in a variety of plant phenotyping tasks, e.g., plant stalk count and stalk width (Baweja et al., 2017), leaf counting in rosette plants (Giuffrida et al., 2018; Ubbens et al., 2018), maize tassel counting (Lu et al., 2017), cotton bloom detection (Xu et al., 2018), wheat spikes detection (Hasan et al., 2018), and rice panicle segmentation (Xiong et al., 2017).

#### 5.1.2. 3D Phenotypes

The plants exhibit increasing architectural complexity with time due to self-occlusions and leaf crossovers, which pose challenges to accurate estimation of 2D component phenotypes, e.g., leaf length, stem angle, and leaf curvature. To overcome these challenges, attempts have been made to reconstruct a 3D model of a plant for accurate estimation of phenotypes. A shape-from-silhouette method is used in Golbach et al. (2016) to reconstruct the 3D model of a tomato seedling from multi-view images. An algorithm for depth imaging-based detection of muskmelon plant for phenotyping in the greenhouse is proposed in Lei et al. (2014a). A detailed review on 3D reconstruction techniques for shoot topology for applications in phenotyping is provided in Gibbs et al. (2016). The method in Liu et al. (2017) uses the structure from motion method to reconstruct the 3D model of a plant using images from multiple side views. The method also deducted the optimal number of images needed for reconstructing a high-quality model. Most cereal crops, e.g., rice, wheat, and maize, have thin ribbon-like architectures with lack of textural surfaces, which limits the success of 3D reconstruction to early growth stages where the distance between the camera and the plant is relatively small facilitating accurate camera calibration.

3D reconstruction of soybean canopies using multisource imaging is presented in Guan et al. (2018). A density-based spatial clustering of applications with noise removal is used to extract canopy information from the raw 3D point cloud. The method in Scharr et al. (2017) uses a voxel-based volume carving for 3D reconstruction of maize shoot to compute leaf-level traits. Depth cameras capture 3D information from plants to segment leaves and reconstruct plant models (Chéné et al., 2012). A semi-automated software pipeline is developed to reconstruct a 3D plant model from the images captured by a depth camera in McCormick et al. (2016). Standard shoot phenotypes such as shoot height, leaf angle, leaf length, and shoot compactness are measured from 3D plant reconstructions to characterize the shoot architecture. The Microsoft Kinect, originally designed for computer gaming environments, has found applications in 3D plant phenotyping in recent times (Chéné et al., 2012; Paulus et al., 2014; Polder and Hofstee, 2014.)

The method in Polder and Hofstee (2014) fuses RGB and depth image captured by Microsoft Kinect sensor to segment muskmelon plants in the greenhouse with cluttered background. The method in Srivastava et al. (2017) provides an algorithm for 3D model reconstruction of wheat plants with occluded leaves, and uses deep learning for drought stress characterization. The remote sensing technology using light detection and ranging (LiDAR) is hypothesized to dominate the future generation of plant phenotyping analysis in outdoor environments Lin (2015). The methods in Sun et al. (2017, 2018) compute morphological traits of the cotton plants, e.g., canopy height, projected canopy area, and plant volume, based on 3D model reconstruction of the plants using top view images captured by LiDAR and real-time kinematic global positioning system (RTK-GPS).

### 5.2. Physiological Phenotypes

A comprehensive review on close proximal assessment of functional dynamics of plants using hyperspectral image analysis is provided in Mishra et al. (2017). Water scarcity causes serious crop losses in agriculture. Global climate change and growing population require research advancement in the understanding of plant resistance mechanisms to drought stress for improved crop yield with minimum resource utilization. The method in Römer et al. (2012) uses a simplex volume maximization technique for early drought stress detection using hyperspectral image sequences of barley plants. Recent developments in hyperspectral imaging for assessment of food quality and safety are discussed in Huang et al. (2014), Lu et al. (2017), and Rungpichayapichet et al. (2017). Hyperspectral image analysis has been used to investigate physiological processes, e.g., determination of salt stress in wheat based on vector-wise similarity measurement (Moghimi et al., 2018), early detection of abiotic stresses (Mohd et al., 2018), disease detection to prevent yield losses (Wahabzada et al., 2016), and early yield prediction (González-Sanchez et al., 2014).

A Matlab based software called SK-UTALCA is introduced in Lobos and Poblete-Echeverría (2017) for applications in plant breeding, precision agriculture, crop protection, ecophysiology plant nutrition, and soil fertility by analyzing high-resolution spectral reflectances. A comprehensive overview of the machine learning techniques in the identification, classification, quantification, and prediction of various biotic and abiotic stress phenotypes are provided in Singh et al. (2016). The method by Raza et al. (2014) combines information extracted from thermal and visible light images, and uses support vector machine (SVM) and Gaussian processes (GP) to identify regions of spinach canopy showing a response to soil water deficit. A review of machine learning approaches including back-propagation neural networks, GP, SVM, rotation forest, CNN and LSTM, for crop yield prediction and nitrogen status estimation in precision agriculture is presented in Chlingaryan et al. (2018).

### 5.3. Temporal Phenotypes

The method in Das Choudhury et al. (2018) uses line graphs to represent the trajectories of component phenotypes, i.e., leaf length, integral leaf-skeleton area, mid-leaf curvature, apex curvature and stem angle, as a function of time in order to demonstrate the genetic influence on the temporal variations of these phenotypes. A novel method for plant emergence detection using adaptive hierarchical segmentation and optical flow based tracking is introduced in Agarwal (2017). The efficacy of the method is demonstrated based on experimental analysis on a dataset consisting of images captured at every 2–5 min intervals starting immediately after planting the seeds.

## 6. Open Problems

Most of the research in image-based plant phenotyping is focused on structural and mainly holistic physiological phenotypes. However, timing determination of important events in the life of a plant, e.g., germination, emergence and senescence of leaves, emergence of flowers and fruits, can provide crucial information about plant's growth and response to biotic and abiotic stresses. Timing detection of such events using computer vision techniques remain an important open problem.

Most of the phenotypes have focused on either aboveground or belowground phenotypes. We propose a new category of phenotype, called integrated phenotype, that establishes relationship between above- and belowground phenotypes affected by abiotic and biotic stresses. Primary root growth is inhibited during P-limitation, and its length determines plant's capability to access stored water in the deeper layers of the soil substratum (Prasad et al., 2012; Paez-Garcia et al., 2015). A new integrated phenotype, e.g., the ratio of stem height to primary root length as a function of time, may be investigated for increased yield of stress tolerant crops by enhancing the capacity of the plant for soil exploration and, thus, water and nutrient acquisition. Algorithms to compute them from plant imagery need to be developed.

Efforts have been made in early detection of stress, e.g., drought and mineral (Kim et al., 2011; Chen and Wang, 2014; van Maarschalkerweerd and Husted, 2015). However, future work is required in the investigation of phenotypes that characterize the prorogation of stress as a function time and also categorize stress into different stages, e.g., slight, moderate, extreme, and exceptional. The speed of recovery from the stresses regulated by genotypes is also an open challenge. The method by Uga et al. (2013) demonstrates that controlling root growth angle contributes to drought tolerance. Hence, we propose a new integrated phenotype to quantify speed of drought recovery from different stress levels in relation to controlled root angle.

Extending the manual leaf tracking method in Das Choudhury et al. (2018), we formulate a new problem called visual growth tracking (i.e., tracking of different parts of an object that grows at different rates over time) using plant image sequences for automated growth monitoring of different components of plants, i.e., leaves, flowers, and fruits.

Computing phenotypes from 2D images (e.g., stem angle) are inherently error-prone as they are dependent on accurate camera views. They are deficient too because the plant is a 3D structure and any projection onto 2D plane results in loss of information. Thus, new and innovative 3D phenotypes, both holistic and component, based on accurate reconstruction of the model of a plant must be developed to accurately characterize its properties. 3D plant model reconstruction has been successful for early growth stages with less architectural complexity (Golbach et al., 2016). Advanced 3D plant model reconstruction algorithms are yet to be developed for entire life cycle of plants covering vegetative and reproductive stages for computation of derived phenotypes, e.g., stem cross-section area as a function of plant height at its different stages, 3D leaf area to leaf length ratio, 3D leaf curvature to leaf length ratio, carbohydrate content of stem with respect to stem volume and plant temperature to convex-hull volume ratio at different stages of stressed plants.

## Author Contributions

SD contributed as the first author in reviewing the literature, compiling the information, preparing the review, and writing the manuscript. AS and TA contributed as co-authors to outline the sections. They critically reviewed the manuscript and provided constructive feedback throughout the process.

### Conflict of Interest Statement

The authors declare that the research was conducted in the absence of any commercial or financial relationships that could be construed as a potential conflict of interest.
